# Open Notes in Mental Health: A Scoping Review of Stakeholder Experiences and Implications for Clinical Practice

**DOI:** 10.3390/healthcare13212777

**Published:** 2025-10-31

**Authors:** Michela Monaci, Setareh Javaher, Serena Barello

**Affiliations:** 1 WHYpsy Lab, Department of Brain and Behavioural Sciences, University of Pavia, 27100 Pavia, Italy; michela.monaci01@universitadipavia.it (M.M.); setareh.javaher01@universitadipavia.it (S.J.); 2 Unit of Applied Psychology, IRCCS Mondino Foundation, 27100 Pavia, Italy

**Keywords:** open notes, mental health, psychiatry, psychotherapy, electronic health records, patient engagement, health literacy, patient-centered medicine, artificial intelligence

## Abstract

**Background/Objectives:** Open Notes—defined as patients’ electronic, portal-based access to clinicians’ narrative documentation within electronic health records (EHRs)—has become routine through policy and portal initiatives. In mental health (MH), transparency intersects with sensitive formulation and risk language, making outcomes contingent on documentation practices, release timing, and reader support. This scoping review mapped empirical evidence on experiences, perceived impacts, and implementation of Open Notes in MH across stakeholders and settings, deriving implications for practice, training, and policy. **Methods:** A PRISMA-ScR-guided review was conducted with a preregistered protocol on OSF. Eligible studies examined Open Notes in MH settings and reported stakeholder perspectives. Two reviewers independently screened and extracted data, analyzed through inductive narrative thematic synthesis. **Results:** Twenty-two studies (2012–2025) from the USA, Sweden, Germany, Canada, and international settings included surveys, qualitative interviews, mixed-methods designs, pilot and quasi-experimental implementations, and a Delphi consensus. Patients consistently reported improved comprehension, recall, empowerment, and—in some cases—greater trust. Large surveys identified error detection and patient-initiated corrections as safety mechanisms, while a minority reported worry or feeling judged by wording. Clinicians adapted documentation—modifying tone, wording, or candor—to minimize misinterpretation. Workload effects were generally modest, limited to occasional clarifications. Implementation and expert studies emphasize organizational readiness, training, patient preparation, and privacy-aware portal design as key enablers of safe transparency. **Conclusions:** In MH, Open Notes function as a communication and engagement tool that strengthens partnership, comprehension, and safety when implemented with attention to risk-sensitive documentation and privacy safeguards.

## 1. Introduction and Background

“Open Notes”—referring specifically to patients’ online access to clinicians’ narrative documentation within electronic health records (EHRs)—has shifted clinical notes from a purely professional artifact to a shared medium of communication in routine patients’ care [1]. Over the past decade, health systems worldwide have increasingly offered patients online access to their medical records through secure portals and apps, often referred to as “open notes.” This practice typically includes visibility of test results, lists of medications, and clinician-written narrative reports. The shift toward giving patients immediate and comprehensive access to their health information reflects a broader emphasis on transparency, patient-centered care, and digital integration in healthcare [2]. As patient portals matured and information-sharing rules expanded, access to visit notes became commonplace across specialties, embedding transparency into everyday practice rather than limiting it to ad-hoc release [3,4]. This shift is consequential for mental health (MH), where documentation interweaves descriptive observation with formulation, provisional hypotheses, risk appraisal, and collateral information—material that is indispensable for continuity and safety but sensitive and context-dependent for lay readers. The mental health domain therefore offers a uniquely informative setting for examining the implications of transparency, as the interpretive and relational nature of clinical notes makes this field particularly sensitive to the communicative, ethical, and therapeutic dimensions of Open Notes. In this environment, the form and language of notes—distinguishing observation from interpretation, signposting uncertainty, and avoiding stigmatizing shorthand—shape how patients infer intent, accuracy, and respect, with implications for the therapeutic relationship and perceived power (un)balance in care encounters [5,6,7].

From a communication perspective, Open Notes functions as an intervention that makes clinicians’ reasoning and plans visible between medical encounters. This visibility has plausible pathways to patient engagement (i.e., patients’ knowledge, information recall, therapeutic adherence) and health literacy (patients’ comprehension and ability to use information). Large multi-system studies outside MH show that most readers understand and trust their notes, while pointing to actionable opportunities to reduce expert jargon and clarify next steps—features central to functional and interactive literacy [3,4]. Early evidence further suggests that patients often feel more engaged in their care plans, gain a clearer understanding of diagnoses and treatments, and develop a stronger sense of trust in their healthcare providers [8]. In many settings, patients report using online access to review or confirm details discussed in consultations, monitor test results, track treatment histories, and facilitate communication with family members or other caregivers [9]. Within MH services, psychotherapy and psychiatry notes can scaffold reflection between sessions and align goals when language is respectful and context for sensitive content is signposted [10].

After Visit Summaries (AVS) sit alongside Open Notes as complementary, action-oriented artifacts: whereas narrative notes preserve clinical reasoning and context, AVS distill next steps, medication changes, and warning signs. Empirically, patients commonly receive and consult AVS delivered via portals, and awareness/use is shaped by portal adoption and behavioral intentions (e.g., attitudes and perceived control) [11]. Readability remains a recurrent barrier—EMR-generated AVS instructions often exceed recommended literacy levels—underscoring the value of plain-language editing and structured layouts [12]. Mixed-methods work in safety-net settings further shows that patients view notes and AVS as complementary: notes supply rationale and detail; AVS clarifies what to do next and how [13]. Design studies indicate that patient-centered AVS (clear headings, concrete action items, simplified wording) are rated more useful by both patients and clinicians, and health-system implementations have identified practical steps to optimize AVS in commercial EHRs [14,15]. Finally, safety literature documents that misalignment between the verbal plan, the note, and the AVS can produce confusion—a remediable risk that strengthens the case for harmonizing these written artifacts.

Despite these benefits, the introduction of open notes poses challenges and potential risks for both patients and clinicians. For patients, the use of medical terminology or unexpected test results may cause confusion or anxiety if not contextualized by a healthcare professional; sensitive information delivered outside of an appointment may create emotional distress; and the discovery of errors or omissions can lead to frustration or mistrust [8]. On the clinician side, concerns include increased workload from patient queries, pressure to rephrase technical language, fear that heightened scrutiny may encourage vague or euphemistic wording, reducing clinical utility and about the impact of being complained about more easily by patients with the data in the medical records [16]. The shift also raises ethical and practical questions about autonomy and equity: while open notes empower patients, those with limited digital literacy or access may be disadvantaged, exacerbating existing inequities [17]. Privacy concerns further arise when unauthorized individuals gain access through shared logins or voluntary credential sharing [18].

Early implementations specific to psychiatry demonstrate feasibility of sharing and foreground practical choices about release timing, mechanisms for clarification, and handling of sensitive information [19,20,21]. The policy landscape has evolved alongside clinical experience: post-mandate surveys describe generally favorable views of note sharing as an engagement tool among clinicians, with concerns concentrated among those reporting greater changes in charting or time burden [22]. Quantitative analyses in other specialties suggest notes became longer yet slightly easier to read after opening, consistent with a shift toward reader-aware documentation [23].

Ethical and practice guidance in MH highlights risk-proportionate governance (narrowly defined exemptions or brief delays when disclosure could cause harm), reader-aware documentation training, and patient-facing orientation materials, as well as usable portals that support questions and corrections: these principles apply to both Open Notes and AVS to ensure consistent written communication [24,25,26,27,28,29,30]. Related studies extend relevance across life stages and roles: in pediatrics, parents in intensive care value access but face linguistic and emotional burdens that call for design and practice supports; in aging, proxy access can enhance involvement but raises issues of digital literacy, consent, and trust [31,32].

New horizons include AI-assisted support. Commentaries and early evaluations propose large language models to help clinicians draft clearer, patient-facing documentation and to help patients interpret notes—opportunities that require strong guardrails for safety and evidentiary grounding [33,34]. For instance, the UK’s National Health Service (NHS) has issued guidance on ambient scribing tools that convert clinician–patient speech into structured documentation [35]. In the UK, a pilot using Microsoft’s Dragon Copilot system has reported reduced documentation burden for clinicians across multiple sites [36]. Internationally, the Hong Kong Hospital Authority is trialling generative-AI tools for medical report writing [37]. While these innovations promise greater efficiency and freed-up clinician time, they also raise important questions around accuracy, liability, and data governance [38]. In parallel, privacy scholarship warns that patients may turn to commercial web services and chatbots to interpret notes, potentially exposing sensitive data; organizational guidance and privacy-preserving design are therefore essential complements to access [39].

Earlier efforts to synthesize emerging evidence were undertaken by [40], who published the first scoping review on sharing clinical notes and electronic health records with people affected by mental health conditions. Their work provided an initial overview of empirical studies and highlighted key ethical and practical challenges. Building on this foundation, the present review expands the evidence base to studies published through 2024, integrating multiple stakeholder perspectives and deriving implications for clinical documentation, training, and policy.

Accordingly, this review aimed to map empirical evidence on Open Notes and shared clinical notes in mental health across stakeholders and settings, with particular attention to patient engagement, health literacy, and the organizational conditions enabling safe and meaningful transparency. To operationalize this aim, the review pursued four specific objectives:

(1) To examine how healthcare managers implement Open Notes in mental health settings, including strategies, challenges, and their impact on patient–provider relationships, documentation practices, and clinical workflows.
(2) To explore how patient portals enabling Open Notes shape technical usability, accessibility, and user experience, as well as patients’ perceived benefits and limitations in mental health care.
(3) To assess how Open Notes influence relational and psychological dimensions of care, particularly patient trust, engagement, and autonomy in mental health, with attention to barriers, facilitators, and gaps in policies for secure and equitable access.
(4) To identify strategies that can support the safe and effective implementation of Open Notes in mental health while addressing privacy, workflow, and equity concerns.

## 2. Materials and Methods

### 2.1. Protocol and Reporting

Methods were specified a priori and preregistered on the Open Science Framework (https://doi.org/10.17605/OSF.IO/TKP3R, accessed on 8 September 2025). This scoping review was conducted in accordance with the Joanna Briggs Institute (JBI) methodology for scoping reviews [41,42] and is reported in line with the Preferred Reporting Items for Systematic Reviews and Meta-Analyses extension for Scoping Reviews (PRISMA-ScR) reporting guideline and checklist [43] (see Supplementary Material S1, Table S1).

### 2.2. Eligibility Criteria

We included peer-reviewed empirical studies published in English that examined Open Notes or patient access to shared clinical notes in MH settings only. Only studies that investigated stakeholder perspectives, experiences, or attitudes (patients, caregivers, clinicians, or organizational leaders) were eligible. Eligible designs were qualitative (e.g., interviews, focus groups), quantitative (e.g., surveys), mixed-methods studies, and pilot or implementation evaluations. We also included expert consensus studies (e.g., Delphi) when specifically focused on Open Notes in MH. Populations included patients/service users, caregivers, clinicians (psychiatrists, psychologists, psychotherapists, nurses, social workers), and MH managers or organizational leaders. The phenomenon of interest was patient-readable clinical notes within electronic health records or patient portals (or closely analogous record-sharing systems). Outcomes of interest included patient–provider relationships, engagement, trust, autonomy, usability, documentation practices, workflow/time, safety/error checking, data security, and policy or ethical considerations.

We excluded secondary research (e.g., reviews, systematic reviews, meta-analyses), non-empirical commentaries or editorials, dissertations, grey literature, and purely technical development papers without stakeholder data. Studies addressing general patient access to health records without explicit reference to Open Notes, or those not reporting stakeholder perspectives, were also excluded. Studies not focused on MH contexts were excluded as well. Full-text availability was required, and no restrictions were applied by year of publication.

### 2.3. Information Sources and Search Strategy

The search strategy combined keywords related to Open Notes and documentation practices (e.g., “open notes,” opennotes, “shared notes,” “clinical documentation,” “patient–clinician documentation,” “sharing clinical notes”), patient-related outcomes and experiences (e.g., “patient engagement,” “patient participation,” perception*, opinion*, preferenc*, experienc*, satisfact*, anxiet*, trust, communication, usability, implementation, outcome*), stakeholder groups (e.g., patient*, clinician*, “healthcare provider*,” “healthcare professional*,” physician*, “clinician–patient relationship”), and MH contexts (e.g., “mental health,” psychiatry, psychiatric, “behavioral health,” psychother*, “mental health service*,” “community mental health,” “forensic mental health,” “child and adolescent mental health,” CAMHS, “mental health professional*”). The search was conducted across four multidisciplinary and health databases (PubMed, Scopus, Web of Science, and CINAHL) from inception to the most recent update. The final search across all databases was completed in August 2025. Search strings were adapted to the indexing and functionalities of each database. In addition, backward citation chasing was performed on reference lists of included studies and relevant networks (e.g., OpenNotes) to capture additional records. Search results were exported for de-duplication and screening. Detailed, database-specific strategies, including full search strings, date stamps, and applied filters (e.g., language limits), are provided in Supplementary Materials (Supplementary Material S2, Table S2).

### 2.4. Selection Process

**Figure 1:**
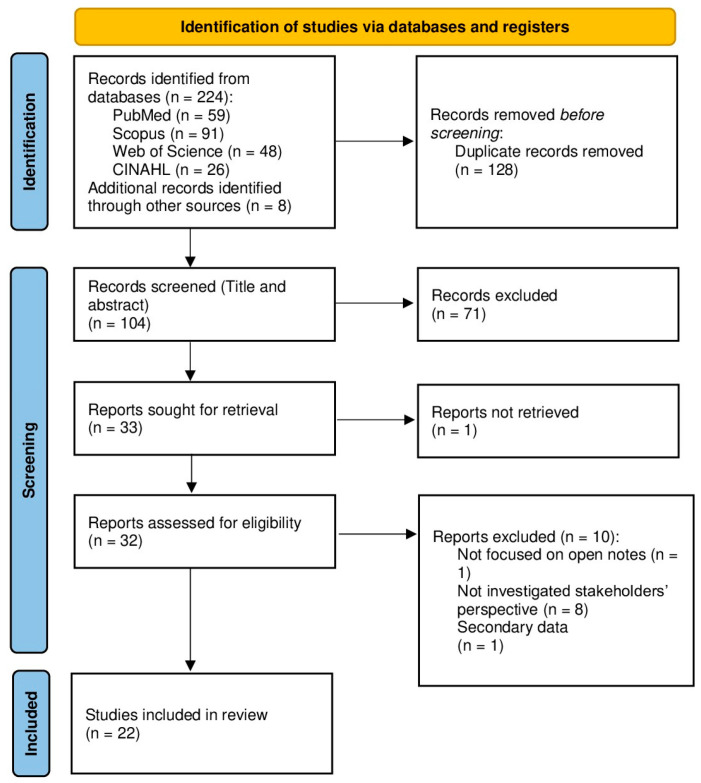
PRISMA flow diagram of study selection and inclusion process.

Following the search, all identified citations were extracted and uploaded into the online systematic review software Rayyan [44], where duplicate entries were removed. Two reviewers (M.M. and S.J.) independently screened titles/abstracts and then full texts against eligibility criteria. Disagreements were resolved through discussion; when necessary, a third reviewer adjudicated. Reasons for exclusion at full text were recorded. The search results and study inclusion process were documented in detail and presented in Figure 1, following the PRISMA-ScR extension guidelines [43,45].

### 2.5. Data Charting (Extraction)

We used a piloted charting form (see Supplementary Material S3, Table S3) to extract authors, year, country/setting, stakeholder (s), design, sample (N) and % female where reported, primary outcomes/measures, and main results. Two reviewers double-charted an initial subset to harmonize interpretation of variables and tags; remaining records were single-charted with verification against the source by a second reviewer when ambiguities arose. When information was missing or unclear (e.g., gender distribution), we recorded “Not Applicable” and did not impute values. Extracted data were subsequently used to inform the thematic synthesis described in Section 2.7.

### 2.6. Synthesis of Results

Consistent with scoping methodology, we conducted a narrative, thematic synthesis. We summarized study characteristics descriptively (counts by country, stakeholder, and design) and aggregated tag frequencies to map prominent topics and concerns. An inductive coding process was applied to identify recurrent ideas and experiences across stakeholder groups. Two reviewers independently coded an initial subset of studies to ensure consistency, refined the coding framework through discussion, and organized themes into higher-order categories that structured the Section 3. Given heterogeneity of outcomes and study designs, no meta-analysis was planned or performed. We did not undertake formal risk-of-bias appraisal, which is optional in scoping reviews; instead, we foreground the breadth of concepts and contexts represented.

### 2.7. Data, Materials, and Code Availability

All materials required to replicate this review are available on OSF under the registration (https://doi.org/10.17605/OSF.IO/TKP3R, accessed on 8 September 2025), including detailed search strategies, screening forms, and data-charting templates, which are also provided in the Supplementary Materials. There are no restrictions on access or reuse beyond standard academic practice.

### 2.8. Ethics

This review synthesizes published studies and does not involve new data collection with human participants or animals; therefore, institutional ethics approval was not required. Ethical approvals for individual primary studies are reported in those publications.

### 2.9. Use of Generative AI

Generative artificial intelligence was used exclusively for language revision (grammar, spelling, punctuation, and minor stylistic edits). All substantive content is the authors’ own work, and the final manuscript was reviewed and approved by the authors.

## 3. Results

### 3.1. Study Selection

The search and screening process followed PRISMA-ScR guidance. In total, records were identified across databases and other sources (*n* = 232), with duplicates removed (*n* = 128). Titles/abstracts screened (*n* = 104) led to full texts assessed for eligibility (*n* = 32). After exclusions for reasons such as “Not focused on Open Notes”, or “Not investigated stakeholders’ perspective”, or “Secondary data” (*n* = 10), studies were included in the review (*n* = 22). The selection pathway is depicted in Figure 1 (PRISMA flow diagram).

### 3.2. Study Characteristics

Twenty-two studies published between 2012 and 2025 were included. Most were conducted in the USA (*n* = 11) [1,4,5,6,7,10,19,46,47,48], followed by Sweden (*n* = 2) [20,21], Germany (*n* = 4) [28,29,32,49], Canada (*n* = 1) [27], Switzerland (*n* = 1) [18], Norway (*n* = 1) [50], and international expert panels (*n* = 2) [25,26].

Study designs comprised quantitative surveys (*n* = 8) [3,4,7,18,29,46], qualitative interviews or focus groups (*n* = 7) [5,6,27,28,32,47,48], mixed-methods (*n* = 3) [10,18,49], pilot/implementation evaluation (*n* = 1) [19], quasi-experimental (*n* = 1) [1], and Delphi consensus (*n* = 2) [26,50].

Stakeholders included patients/service users (*n* = 5) [3,4,6,10,46], clinicians & nurses (*n* = 4) [5,7,27,49], clinical social workers (*n* = 1) [48], psychotherapists or psychotherapy students/trainees (*n* = 2) [18,29], psychiatrics and psychiatric care professionals in Swedish services (*n* = 3) [20,21,28], and experts/decision-makers (*n* = 3) [25,26,50]. Four studies included both patients and clinicians [1,19,32,47].

Sample sizes ranged from small, in-depth qualitative cohorts (*n* ≈ 11–28) to very large surveys (*n* ≈ 64–28,782). Among studies reporting gender, the mean proportion of female participants was ~71.9% (range 21–90%). Full study characteristics, including aims, outcomes, and key results, are presented in Table 1.

### 3.3. Thematic Synthesis (Across Outcomes)

Across the corpus, effects are clustered into a small set of reproducible domains. Documentation change and patient engagement were the most prevalent themes (*n* = 12), followed by safety risk (*n* = 9), patient worry/distress (*n* = 8), error checking (*n* = 5), policy and privacy (*n* = 4), trust (*n* = 6), and usability/readability (*n* = 5).

On the patient side, Open Notes in MH was consistently associated with better comprehension and recall, greater sense of control, and perceived empowerment, with gains in trust when written accounts were accurate, respectful, and aligned with the clinical encounter [3,4,10,46,47]. Large multi-site surveys also documented error detection and correction as a safety mechanism [3,4,18]. However, a non-trivial minority reported worry, feelings of being judged, or confusion when encountering uncontextualized hypotheses or risk language [6,10,46]. Recent studies expanded these observations to specific groups, such as adolescents and older adults, highlighting the need for developmental tailoring and attention to privacy, proxy access, and contextual explanation when notes are shared [32,50].

On the clinician side, multiple studies—across the U.S. Veterans Health Administration, Swedish psychiatry, and German practice—described systematic adaptations in documentation (calibrated wording/tone, and in some cases reduced candor) to pre-empt misunderstanding or harm [5,7,20,21,28,48]. Post-implementation surveys indicated that apprehensions about misinterpretation persisted, even though visit length generally did not increase; operational burden was concentrated in occasional clarifications rather than routine workflow expansion [21,27]. Further data underline that usability barriers and documentation workload remain significant implementation concerns [49].

Implementation-focused studies and expert consensus specified moderators and enablers—including diagnosis and acuity, readability and portal usability, time for writing or debrief, and clear legal/privacy rules—along with proportionate exemptions or delays for high-risk scenarios and training/patient-preparation to support risk-sensitive, patient-centred documentation [25,26,29]. The importance of governance clarity and clinician support was also emphasized for adolescent and geriatric contexts, where proportional access and tailored preparation are key [32,50].

In aggregate, the pattern is scientifically coherent with Open Notes operating as a communication intervention: enabling engagement and safety through error checking when documentation is reader-aware and supports are present, while elevating distress and misinterpretation risks when those conditions are absent, with net workload effects modest on average. Beyond clinician and patient experiences, several studies [19,20,21,25,26,27,28] addressed the managerial and organizational dimension of Open Notes implementation. Healthcare managers and leaders were reported to influence documentation practices, workflow adaptation, and the development of policies for proportionate exemptions and patient preparation. Organizational readiness—encompassing training provision, governance clarity, and privacy-aware portal design—was consistently identified as a precondition for sustainable transparency in mental health care.

### 3.4. Findings by Stakeholder Group

*Patients.* Patient-focused studies, both qualitative and survey-based, consistently reported benefits in comprehension, recall, and perceived control, with trust reinforced when notes were experienced as accurate, respectful, and aligned with encounters [3,10,46,47]. Large multi-site surveys highlighted error detection and patient-initiated corrections, positioning patients as contributors to safety [3,4,18]. Reported harms—affecting a minority—were linked to worry, offense, or feelings of being judged by note formulations [10,46]. Trust deteriorated when patients perceived errors, omissions, or judgmental language [6]. Recent work has extended these findings to adolescent and older adult populations, highlighting that preparatory discussion, privacy safeguards, and accessibility support are essential for safe and meaningful engagement [32,50].

*Clinicians, Psychiatrists, and Psychotherapists.* Studies with clinicians documented shifts in documentation practices in response to anticipated patient readership, including more careful phrasing, avoidance of stigmatizing terminology, and occasional reduction in candor [5,7,48]. Swedish pre-/post-implementation surveys showed persistent concerns about misunderstandings and conflicts, with little evidence of extended visit length [20,21]. Canadian interviews emphasized the need for training, patient preparation, and clear policies for handling sensitive information [27]. In Germany, psychotherapists expressed negative expectations regarding therapeutic impact and workload [29], while psychiatrists underscored enabling conditions for safe implementation—triage by diagnosis/acuity, adequate time and resources, integrated portals, and robust legal/privacy frameworks—anticipating both benefits (transparency, trust) and risks (distress, misunderstanding) [28,49].

*Experts and Implementation Leaders.* International expert consensus and inquiry converged on pragmatic implementation strategies: training clinicians in patient-centred, risk-sensitive documentation; providing patient-facing preparation resources; applying proportionate exemptions or brief delays in high-risk scenarios; and ensuring policy and privacy clarity supported by usable, accessible patient portals [25,26]. A subsequent Delphi consensus expanded these recommendations to adolescent mental health care, offering operational guidance on clinician support, graduated disclosure, and criteria for withholding sensitive information when clinically justified [50].

### 3.5. Contextual and Implementation Factors

Variation in reported outcomes appeared to be influenced by diagnosis and acuity, care setting (inpatient, partial hospital/day clinic, outpatient), documentation style, and portal usability. German interviews emphasized the need for sufficient time for writing and debrief, integrated patient portals, and legal/data-protection clarity, while subsequent evidence highlighted persistent usability limitations and workflow misalignment as additional barriers to sustained adoption [28,49]. Swedish pre-/post-implementation surveys indicated that even after roll-out, clinicians’ concerns about misinterpretation persisted, alongside subtle shifts in documentation tone [20,21]. Canadian interviews similarly underscored the value of training, policy guidance, and preparation resources for safe implementation [27]. In adolescent care, structured clinician support and communication framing have been recommended to prevent misunderstanding [50], while in older-adult settings, privacy and caregiver access protocols remain key implementation priorities [32]. Across contexts, workload effects were generally modest, though some clinicians reported occasional additional clarification [21,27,48].

### 3.6. Summary of Effects on Safety and Workflow

The safety potential of Open Notes in MH was most evident in patient error detection and follow-up contacts to correct inaccuracies, which large surveys and recent studies identified as a meaningful safeguard [3,4,18]. The principal risks arose from patient distress and misinterpretation of clinical language, which in turn influenced clinicians’ documentation practices [5,7,10,20,21,46]. Emerging evidence further suggests that these effects vary by population, with adolescent and geriatric groups benefiting most when supported by clear communication and privacy-oriented design, while clinician safety perceptions depend on usability and workflow alignment [32,49,50]. Overall, workflow effects were described as limited, with local increases linked primarily to clarification needs. Thematic counts and per-study details are summarized in Table 1, with full extraction fields (including sample sizes and gender distribution) available in the Supplementary Materials (Table S3).

## 4. Discussion

Viewed through the lens of the broader literature, Open Notes in mental health (MH) functions as a communication intervention that can support engagement and health literacy while reconfiguring documentation practice. Focusing on MH provides a particularly informative case, as mental health documentation involves interpretive and relational dimensions that are especially sensitive to transparency. Clinicians’ notes in this context often integrate descriptive and diagnostic formulations, making the field distinctive for examining how openness affects communication, trust, and therapeutic dynamics. Patient-reported benefits—improved recall, preparedness, and a sense of partnership—are conceptually aligned with multi-system findings outside MH showing high comprehensibility and perceived accuracy, alongside requests for less jargon and clearer action items [2,3,4,8,9]. In MH specifically, patients emphasize respectful tone, alignment with the clinical encounter, and adequate context for sensitive content; when these elements are absent, a non-trivial minority report worry or feeling judged [10,46,47]. Qualitative work on trust underscores this asymmetry: accuracy and respect foster trust, whereas perceived errors or judgmental phrasing erode it [6]. Similar patterns have been reported among adolescent and older-adult populations, underscoring the importance of clinician guidance and privacy safeguards [32,50]. The evidence also points to the central role of healthcare managers and organizational leadership in shaping successful implementation. Across contexts (Canada, Sweden, Germany, and international expert panels), leadership engagement and structured implementation strategies were key to fostering clinician acceptance and aligning documentation practices with patient-centered values [20,21,26,27,28]. Leadership decisions regarding training time, documentation standards, and privacy governance emerged as critical enablers of clinician confidence and sustainable adoption of Open Notes. Although most studies did not disaggregate findings by level of care, the reported benefits of Open Notes—such as improved engagement, trust, and shared understanding—were consistently observed across hospital, outpatient, and community mental health settings.

On the clinician side, adaptive documentation change—calibrating wording and tone and occasionally reducing candor—appears across systems and professional roles [5,7,20,21,28]. Rather than implying inevitable degradation of the clinical record, convergent data suggest a shift toward reader-aware specificity; in oncology, for example, progress notes became longer yet slightly easier to read after opening, a pattern consistent with the MH narrative [23]. Evidence from German mental health services similarly highlighted usability and workflow barriers as relevant contextual factors [49]. Early post-mandate surveys indicate that favorable attitudes co-occur with perceived utility for engagement, whereas less favorable views are associated with greater charting changes and time burden [22]. Collectively, these findings reinforce that implementation choices—documentation training, patient orientation, portal design, and proportionate timing/exemptions—shape experience and workload as much as professional disposition [8,16,24,25,26,27].

Treating documentation as reader-facing communication also extends to After Visit Summaries (AVS). Practical levers include: (1) harmonizing AVS with the narrative note so that “what to do next” aligns with documented reasoning; (2) applying patient-centred AVS patterns (topic headers, concrete action lists, medication tables, contact details) that have been associated with higher perceived usefulness; and (3) enforcing readability targets given that EMR-generated AVS frequently exceed recommended reading levels [11,12,14,15]. When AVS–note congruence is weak, case reports show avoidable confusion; conversely, redesign projects demonstrate feasible, scalable improvements within commercial EHRs [13,15,51].

Context-specific literature broadens the implementation lens. In pediatric critical care, parents derive applied and emotional benefits from access but encounter linguistic and role-boundary burdens, highlighting the need for orientation materials and coordinated team messaging in high-acuity environments [31]. Among older adults and care partners, proxy access can deepen shared understanding yet depends on digital literacy, consent practices, and trust; psychiatric services should therefore attend to equitable access configurations and clear consent workflows [17,32]. Emerging practice guidance provides concrete levers: patient-centered documentation principles—plain language, respectful descriptors, explicit separation of observation from interpretation, and brief summaries—translate ethical commitments into operational habit [30].

Beyond individual and organizational practices, several studies also underscored the importance of safeguarding privacy and ensuring that digital infrastructures are adequately prepared to support secure and transparent documentation in mental health care.

The forward look includes both opportunity and caution around AI-assisted supports. Expert viewpoints argue that large language models may help clinicians craft clearer, empathic notes and help patients interpret them; early evaluations show usefulness is sensitive to prompt design, with evidentiary grounding an explicit weakness requiring governance and human oversight [33,34]. In parallel, privacy analyses caution that patient use of commercial web tools to interpret notes may expand data exposure, urging organizations to provide safer interpretive resources and privacy-preserving design [39]. Together, these strands point toward a pragmatic synthesis: treat the clinical note as reader-facing communication; pair access with training, orientation, and proportionate exemptions; optimize portals for questions and corrections; and evaluate supportive technologies with attention to evidence, empathy, and privacy [18,24,25,26,27,28,29,32,49,50].

Taken together, these findings indicate that Open Notes represent a practical pathway toward more transparent, collaborative, and patient-centered mental health care.

### Limitations

This review should be interpreted in light of several limitations of the underlying evidence. Most studies were conducted in high-income systems (U.S., Sweden, Germany, Canada), many relied on cross-sectional self-report, and outcomes were heterogeneous, precluding meta-analysis [3,4,6,7,46,48]. Objective system-level outcomes (e.g., admissions, incident reports temporally linked to note access) remain under-studied, and some stakeholder groups—youth, families, and community MH teams—are under-represented. No formal quality or risk-of-bias appraisal of the included studies was conducted, as the aim of this review was to map the breadth and nature of available evidence rather than to evaluate its methodological rigor. Nevertheless, the absence of such assessment limits the extent to which the robustness of individual studies can be judged. Nonetheless, triangulation across methods and settings, plus alignment with expert consensus, strengthens confidence in the main inferences [25,26].

Future investigations are encouraged to complement this mapping with theoretically informed and experimental studies aimed at elucidating the mechanisms through which Open Notes affect engagement, trust, and safety in mental health care. Moreover, building on this evidence base, future studies should include empirical and simulation-based analyses to evaluate how variations in system design, implementation strategies, and security protocols influence both the performance and safety of Open Notes platforms in mental health care. Priority targets include randomized or stepped-wedge evaluations of documentation training and patient-preparation packages; development and validation of readability and respectfulness metrics for notes and AVS; and analyses that stratify by diagnosis/acuity and setting (inpatient, day hospital, outpatient) to test the conditionality suggested by qualitative work [28,29]. Mixed-methods designs that link objective outcomes (e.g., error-correction rates, incident reports, service use) with patient- and clinician-reported experiences would help resolve uncertainties about net safety and workload effects [3,4,18,27,47]. Co-design with people with lived experience can refine language guidance, patient materials (including AVS), and portal features to maximize clarity and minimize unintended harms.

## 5. Conclusions

Open Notes in mental health should be understood as a scalable communication intervention, not merely a technical policy. When transparency is paired with risk-sensitive, patient-centred documentation, brief patient preparation, proportionate exemptions for narrowly defined high-risk scenarios, and usable, privacy-aware portals, patients commonly report better engagement and recall, and many describe strengthened trust; Open Notes can also contribute to safety through patient-initiated error checking [3,4,8,9,10,46,49,50]. The principal risks—patient distress and defensive documentation—are real but manageable with targeted supports, clear governance and continuous patient involvement [5,7,21,27,28,29,32,48].

Effective implementation also depends on organizational readiness and workflow adaptation, ensuring that documentation systems, training, and digital interfaces support both clinicians and patients. Open Notes reframes the clinical note as a tool to promote clinician–patient communication and a trusting relationship. Language choices—plain wording, respectful tone, and explicit separation of observation from interpretation—are not cosmetic; they are therapeutic tools that shape comprehension, emotion, and alliance. Treating documentation as a reader-facing artifact invites co-production: clinicians can contextualize risk statements, link plans to patient goals, and incorporate the patient’s own formulations; patients, in turn, can review, reflect, and provide corrections or preferences. Framed this way, Open Notes becomes a practical pathway toward more patient-oriented and personalized care, supporting health literacy, shared decision-making, and continuity between visits [3,4,10,47].

As systems move toward broader digital access, attention to usability, privacy, and equity will determine whether transparency translates into meaningful and safe participation in care. Health systems that move beyond simple access toggles—by investing in documentation training, brief orientation materials for patients, clear exemption policies, and feedback channels for questions and corrections—can translate transparency into safer, more participatory, and more personalized mental health care [8,16,18].

In addition to individual documentation practices, organizational and leadership engagement is essential for translating transparency mandates into routine mental health care. Healthcare managers can operationalize Open Notes through structured training, resource allocation, and policies that balance openness with safety and privacy. Evidence from several studies [20,21,25,26,27,28] demonstrates that leadership commitment and governance clarity are decisive for sustaining Open Notes as a safe and meaningful component of patient-centered mental health services [32,50].

## Figures and Tables

**Table 1:** Characteristics of included studies and main results.

Study	Year	Main Aim	Country/Setting	Stakeholder(s)	Design	Sample (N)	Female (%)	Primary Outcomes/Measures	Main Results
Blease et al., 2021 [25]	2021	Elicit international expert recommendations to prepare patients and clinicians for MH OpenNotes.	International (6 countries)	Experts (clinicians, leaders, advocates)	Qualitative expert inquiry	70	50.0	Recommendations for preparing patients and clinicians for MH OpenNotes (training, communication, exceptions, safety).	Consensus guidance emphasized proactive patient education, clinician training on language, clear exemption pathways for safety risks, and organizational policies for consistent implementation.
Blease et al., 2021 [26]	2021	Build expert consensus on benefits, harms, and mitigations of Open Notes in mental health.	International	Experts	Three-round modified Delphi	70	50.0	Consensus on benefits and harms of MH OpenNotes; prioritization of risks/mitigations.	Top benefits: engagement, accuracy checks, recall, safety through error detection. Top harms: increased worry/confusion, potential impact on therapeutic candor; mitigation through guidance and training.
Chimowitz et al., 2020 [48]	2020	To examine clinical social workers’ attitudes and experiences with sharing psychotherapy notes via OpenNotes	USA (BIDMC; large urban academic medical center + community health center, Boston)	Clinical social workers (therapists, MSWs)	Qualitarive interviews and focus group	9	NA	Experiences, concerns, and perceived benefits of open psychotherapy notes	Participating therapists reported mostly positive attitudes, minimal workload disruption, and potential benefits for communication and patient empowerment, though noted patients rarely discussed notes. Non-participating therapists expressed concerns about privacy, workload, clinical language misinterpretation, and potential harm to therapeutic relationships. Overall, sharing notes was feasible and often beneficial, but more training and research are needed
Cromer et al., 2017 [6]	2017	Examine how reading mental health notes influences veterans’ trust in their clinicians.	USA (VA)	Patients	Qualitative interviews	28	57.0	How reading mental health notes influences trust in clinicians.	Trust frequently increased due to perceived honesty/consistency; decreases occurred when patients perceived errors, judgmental language, or discrepancies.
Delbanco et al., 2012 [1]	2012	Evaluate effects of inviting patients to read primary care visit notes on patients and clinicians (pre/post).	USA (3 health systems)	Primary care physicians & adult patients	Quasi-experimental (pre/post surveys)	Patients with ≥1 note: 13,564; post-survey ≈ 5391	NA	Patient-reported effects on understanding, self-care, medication adherence; clinician-reported workload, visit length, and documentation changes.	Patients widely reported benefits (better understanding, feeling in control); clinicians reported minimal change in workload or visit length but some changed wording; support for continued access was high.
Denneson et al., 2017 [5]	2017	Explore how patient access to MH notes affects clinicians’ perceptions of power, documentation, and the therapeutic relationship.	USA (VA)	Clinicians & Nurses	Qualitative interviews	28	57.0	Perceived impact of patients’ online access to mental health notes on power dynamics, documentation practices, and therapeutic alliance.	Clinicians described a shift in power toward patients and increased attention to wording; some limited candor in notes to prevent harm, but many recognized transparency and engagement benefits.
Denneson et al., 2018 [46]	2018	Quantify patients’ positive and negative reactions to reading their mental health notes online.	USA (VA)	Patients (Veterans)	Cross-sectional survey of note readers	178	21.0	Patient-reported positive (e.g., feeling informed, trusting) and negative (e.g., worry, offense) reactions to reading MH notes; impacts on care use and self-management.	Most respondents reported positive effects on understanding and involvement; a minority reported worry or feeling judged; overall net positive balance with actionable feedback for clinicians.
Dobscha et al., 2016 [7]	2016	Assess mental health clinicians’ attitudes, perceived benefits/risks, and self-reported documentation changes with OpenNotes in VA.	USA (single VA Medical Center)	Clinicians & Nurses	Cross-sectional survey	208	56.3	Attitudes toward OpenNotes in mental health; perceived benefits/harms; self-reported changes to documentation; effects on workflow & therapeutic relationship.	About half endorsed OpenNotes as a good idea; many reported writing notes less candidly or changing wording; perceived risks included patient worry and safety concerns, but most anticipated benefits for engagement and accuracy checks.
Kassam et al., 2022 [27]	2022	Explore Canadian psychiatric clinicians’ experiences, uses, benefits, and challenges with OpenNotes.	Canada (Ontario psychiatric settings)	Clinicians	Qualitative interviews	23	82.6	Perceived uses, benefits, and challenges of OpenNotes in psychiatric care; implementation supports needed.	Clinicians supported transparency and collaboration but cited need for training, patient preparation, and clear policies for sensitive content and safety concerns.
Kharko et al., 2024 [18]	2024	Explore psychotherapy trainees’ familiarity with and opinions about patients’ access to psychotherapy notes (“open notes”)	Switzerland (University of Basel psychotherapy training programs)	Psychotherapy students/trainees (various programs)	Mixed-methods survey (web-based questionnaire with quantitative and qualitative components)	72	86.1	Psychotherapy trainees’ familiarity with open notes and their opinions on the impact for patients, therapists, and psychotherapy practice	Views were mixed: many trainees anticipated negative impacts (increased workload, constrained note-writing, risk to therapeutic alliance, patient confusion/offense) but also saw benefits (greater transparency, trust, empowerment, better informed consent, communication). Four main themes: negative impact on therapy, positive impact on therapy, impact on patients, and documentation changes. Almost all (94.1%) agreed education on open notes should be part of training
Leveille et al., 2020 [4]	2020	Assess patient evaluations (understandability, accuracy, helpfulness, safety) for a single recently read visit note.	USA multi-site (general)	Patients (general; note readers)	Cross-sectional survey focused on a single recalled note	22,947	NA	For one recent note: understandability, correctness, helpfulness, and safety (error identification); comparisons across note types.	Vast majority rated notes easy to understand and accurate; many reported finding errors/omissions and contacting clinicians; mental health notes represented a small fraction but showed similar usability concerns.
Meier-Diedrich et al., 2025 [32]	2025	Examine objective and perceived changes in clinical documentation after implementing Open Notes in mental health care.	Germany/Psychiatric outpatient clinics (Brandenburg Medical School, Rüdersdorf)	Clinicians (psychiatrists, psychologists, social worker)	Mixed-methods pre-post study (linguistic analysis + qualitative interviews)	107 (97 patients; 10 clinicians)	55.7 (patients); 50.0 (clinicians)	Linguistic analysis of 16 language features; qualitative interviews on documentation practices and perceptions.	After Open Notes, notes showed significantly increased comprehensible, resource-oriented, personal, and positive emotional language and reduced controlling, demeaning, and stigmatizing language. Clinicians wrote more patient-friendly and reflective notes, with greater attention to clarity and sensitivity; workload increased but no loss of clinical content was reported.
Meier-Diedrich et al., 2025 [49]	2025	Explore the experiences, barriers, and opportunities of older mental health patients and their care partners using proxy access to Open Notes.	Germany/Psychiatric outpatient clinics (Rüdersdorf and Strausberg)	Older patients (≥60 years) and care partners (family members, spouses, children)	Qualitative interviews (constructivist grounded theory)	20 (10 patients; 10 care partners)	70	Experiences, digital literacy, trust, relationship dynamics, autonomy, and perceived impact of proxy Open Notes.	Access to Open Notes with proxy accounts improved understanding, engagement, and communication between patients, care partners, and clinicians; benefits included better preparation for appointments and enhanced support, but also raised concerns about privacy, control, and emotional burden; trust and digital literacy were key enablers.
Nielsen et al., 2024 [50]	2024	Develop expert-based recommendations for digital sharing of clinical notes with adolescents in mental health care and assess staff agreement with these recommendations.	Norway/Child and Adolescent Mental Health Services (CAMHS)	Experts (authors of scientific papers) and CAMHS staff	Delphi consensus + cross-sectional survey	Delphi: 27 (Round 1)/21 (Round 2); Staff survey: 41	81 (Delphi); 90 (Staff)	Consensus level on 43 proposed recommendations; staff agreement with 17 finalized recommendations.	Consensus reached on 17 recommendations covering how to introduce access, write notes, support professionals, and withhold notes when necessary. High agreement among staff (>60%) on all recommendations, especially training, respectful language, and clear criteria for withholding. Uncertainty remained on optimal age and parental access timing.
O’Neill et al., 2019 [10]	2019	Investigate psychotherapy patients’ experiences reading their therapists’ notes and perceived impacts on care.	USA (BIDMC outpatient psychotherapy)	Patients	Mixed-methods (survey + 11 interviews)	85 survey; 11 interviews	76.0	Perceived helpfulness, control, trust, self-care; adverse reactions; interest in continued access to psychotherapy notes.	Large majority valued access for reflection, recall, and trust-building; few adverse experiences were reported; most wanted continued access.
Peck et al., 2017 [19]	2017	Pilot patient access to electronic psychiatric records and assess feasibility, satisfaction, and note characteristics.	USA (Beth Israel Deaconess, outpatient psychiatry)	Patients & Clinicians	Pilot with post-visit surveys & note ratings	64 (52 patients; 12 clinicians; 160 notes rated)	NA	Feasibility; patient satisfaction/understanding; clinician perceptions; note readability/appropriateness.	Most patients reported satisfaction and better recall; clinicians generally accepted feasibility but flagged potential for confusion and extra clarification; few instances of perceived harm in rated notes.
Petersson & Erlingsdóttir, 2018 [20]	2018	Assess psychiatric professionals’ expectations and concerns before implementation of Open Notes in Swedish psychiatric care.	Sweden (regional psychiatric services)	Psychiatric care professionals	Pre-implementation web survey	871	73.8	Expected impacts of open notes on patients, documentation, workflow, and therapeutic relationship (before roll-out).	Many anticipated more patient worry and disagreements and reported intent to be less candid in documentation; anticipated limited benefits for patient involvement relative to somatic care.
Petersson & Erlingsdóttir, 2018 [21]	2018	Evaluate psychiatric professionals’ experiences and perceived impacts after Open Notes implementation in Sweden.	Sweden (regional psychiatric services)	Psychiatric care professionals	Post-implementation web survey	699	72.1	Experienced impacts on documentation practices, workflow, patient contact, and patient understanding after roll-out.	Concerns about reduced candor persisted; little change in visit length; some professionals perceived increased patient involvement, but apprehensions about misunderstandings and conflicts remained.
Pisciotta et al., 2019 [47]	2019	To provide mental health clinicians with recommendations identified by patients and clinicians for practicing effectively in the context of OpenNotes	USA (Veterans Health Administration -Pacific Northwest, 13 urban and rural facilities)	Patients (veterans) & Clinicians	Qualitative interviews	56 (28 patients; 28 clinicians)	57.0	Identification of recommendations regarding note-writing, communication, and use of notes	Three domains: (1) write respectful, clear notes highlighting progress; (2) communicate with patients about notes (transparency, openness to discussion); (3) use notes as a therapeutic resource and collaborative tool
Schwarz et al., 2024 [28]	2024	Identify conditions and perceived consequences for implementing Open Notes in psychiatry from psychiatrists’ perspectives.	Germany (adult psychiatry; inpatient, day clinic, outpatient)	Psychiatrists	Qualitative semi-structured interviews; thematic analysis (COREQ)	18	44.4	Perceived conditions for successful implementation (user groups/settings, time, resources/usability, legal/privacy) and expected changes (documentation, treatment processes, clinician–patient interaction).	Four conditions needed (diagnoses/severity triage; more time for writing/debrief; simple integrated portal & resources; clear legal/data-protection rules). Expected changes: more patient-centred language and occasional ‘closed’ notes; potential benefits (transparency, trust, continuity, adherence) but risks (misunderstandings, distress, conflicts) especially in acute phases—suggest case-by-case access limits.
Schwarz et al., 2024 [29]	2024	Describe German psychotherapists’ views and expectations regarding OpenNotes in psychotherapy practice.	Germany (nationwide online survey)	Psychotherapists (medical & psychological)	Cross-sectional online survey	129	62.0	Attitudes and expectations about OpenNotes; anticipated effects on psychotherapy, documentation burden, and relationship.	Predominantly negative expectations regarding therapeutic impact and workload; respondents requested clearer exemptions and patient preparation.
Walker et al., 2019 [3]	2019	Characterize patient experiences and perceived impacts after several years of exposure to OpenNotes across health systems.	USA multi-site (BIDMC, UW Medicine, Geisinger)	Patients (general)	Large cross-sectional survey	28,782	62.8	Reading frequency; perceived helpfulness; effects on understanding, recall, engagement, and safety-checking; communication with clinicians.	Most readers reported better understanding/recall and feeling in control; many found notes very helpful for managing care and confirming accuracy; small minority reported confusion or worry.

MH = Mental Health; NA = Not Applicable; VA = Veterans Affairs; CAMHS = Child and Adolescent Mental Health Services; COREQ = Consolidated Criteria for Reporting Qualitative Research; BIDMC = Beth Israel Deaconess Medical Center; UW = University of Washington Medicine.

## Data Availability

No new data were created or analyzed in this study. Data sharing is not applicable to this article.

## Supplementary Materials

The following supporting information can be downloaded at: https://www.mdpi.com/article/10.3390/healthcare13212777/s1. Supplementary Material S1: Table S1. PRISMA checklist; Supplementary Material S2: Table S2. Search strategy; Supplementary Material S3: Table S3. Data extraction form.

## Abbreviations

The following abbreviations are used in this manuscript:

AI	Artificial Intelligence
AVS	After-Visit Summaries
EHR	Electronic Health Record(s)
JBI	Joanna Briggs Institute
MH	Mental Health
OSF	Open Science Framework
PRISMA-ScR	Preferred Reporting Items for Systematic Reviews and Meta-Analyses extension for Scoping Reviews
VA	Veteran Affairs
