# Supplementary material for: Open Notes in Mental Health: A Scoping Review of Stakeholder Experiences and Implications for Clinical Practice
Source: Healthcare (Basel). 2025 Oct 31;13(21):2777. doi: 10.3390/healthcare13212777 (PMC12607305; doi:10.3390/healthcare13212777)
Supplement: Supplementary file 1 [file healthcare-13-02777-s001.zip › Supplementary material 2.pdf]

Supplementary material 2

Table S2. Search strategy

Database	Query	Records identified
PubMed	(((("open notes"[Title/Abstract] OR opennotes[Title/Abstract] OR "shared notes"[Title/Abstract] OR "clinical documentation"[Title/Abstract] OR "patient-clinician documentation"[Title/Abstract] OR "sharing clinical notes"[Title/Abstract])) AND (("patient engagement"[Title/Abstract] OR "patient participation"[Title/Abstract] OR perception*[Title/Abstract] OR opinion*[Title/Abstract] OR preferenc*[Title/Abstract] OR experienc*[Title/Abstract] OR satisfact*[Title/Abstract] OR anxiet*[Title/Abstract] OR trust[Title/Abstract] OR communication[Title/Abstract] OR usability[Title/Abstract] OR implementation[Title/Abstract] OR outcome*[Title/Abstract]))) AND ((patient*[Title/Abstract] OR clinician*[Title/Abstract] OR "healthcare provider*"[Title/Abstract] OR "healthcare professional*"[Title/Abstract] OR physician*[Title/Abstract] OR "clinician-patient relationship"[Title/Abstract]))) AND (("mental health"[Title/Abstract] OR psychiatry[Title/Abstract] OR psychiatric[Title/Abstract] OR "behavioral health"[Title/Abstract] OR psychother*[Title/Abstract] OR "mental health service*"[Title/Abstract] OR "community mental health"[Title/Abstract] OR "forensic mental health"[Title/Abstract] OR "child and adolescent mental health"[Title/Abstract] OR CAMHS[Title/Abstract] OR "mental health professional*"[Title/Abstract]))	59
Scopus	(TITLE-ABS-KEY (("open notes" OR opennotes OR "shared notes" OR "clinical documentation" OR "patient-clinician documentation" OR "sharing clinical notes")) AND TITLE-ABS-KEY (("patient engagement" OR "patient participation" OR perception* OR opinion* OR preferenc* OR experien c* OR satisfact* OR anxiet* OR trust OR communication OR usability O R implementation OR outcome*)) AND TITLE-ABS-KEY ((patient* OR clinician* OR "healthcare provider*" OR "healthcare professional*" OR physician* OR "clinician-patient relationship")) AND TITLE-ABS-KEY (("mental health" OR psychiatry OR psychiatric OR "behavioral health" OR psychother* OR "mental health service*" OR "community mental health" OR "forensic mental health" OR "child and adolescent mental health" OR CAMHS OR "mental health professional*")))	91

Web of Science	("open notes" OR opennotes OR "shared notes" OR "clinical documentation" OR "patient-clinician documentation" OR "sharing clinical notes") (Abstract) and ("patient engagement" OR "patient participation" OR perception* OR opinion* OR preferenc* OR experienc* OR satisfact* OR anxiet* OR trust OR communication OR usability OR implementation OR outcome*) (Abstract) and (patient* OR clinician* OR "healthcare provider*" OR "healthcare professional*" OR physician* OR "clinician-patient relationship") (Abstract) and ("mental health" OR psychiatry OR psychiatric OR "behavioral health" OR psychother* OR "mental health service*" OR "community mental health" OR "forensic mental health" OR "child and adolescent mental health" OR CAMHS OR "mental health professional*") (Abstract)	48
CINAHL	XB (("open notes" OR opennotes OR "shared notes" OR "clinical documentation" OR "patient-clinician documentation" OR "sharing clinical notes")) AND XB (("patient engagement" OR "patient participation" OR perception* OR opinion* OR preferenc* OR experienc* OR satisfact* OR anxiet* OR trust OR communication OR usability OR implementation OR outcome*)) AND XB ((patient* OR clinician* OR "healthcare provider*" OR "healthcare professional*" OR physician* OR "clinician-patient relationship")) AND XB (("mental health" OR psychiatry OR psychiatric OR "behavioral health" OR psychother* OR "mental health service*" OR "community mental health" OR "forensic mental health" OR "child and adolescent mental health" OR CAMHS OR "mental health professional*"))	26
