# Supplementary material for: Open Notes in Mental Health: A Scoping Review of Stakeholder Experiences and Implications for Clinical Practice
Source: Healthcare (Basel). 2025 Oct 31;13(21):2777. doi: 10.3390/healthcare13212777 (PMC12607305; doi:10.3390/healthcare13212777)
Supplement: Supplementary file 1 [file healthcare-13-02777-s001.zip › Supplementary material 3.pdf]

Supplementary material 3

Table S3. Data extraction form

**Study**	**Year**	**Main aim**	**Country/Setting**	**Stakeholder(s)**	**Design**	**Sample (N)**	**Female (%)**	**Primary outcomes/measures**	**Main results**
